# Agomelatine, Ketamine and Vortioxetine Attenuate Energy Cell Metabolism—In Vitro Study

**DOI:** 10.3390/ijms232213824

**Published:** 2022-11-10

**Authors:** Matej Ľupták, Zdeněk Fišar, Jana Hroudová

**Affiliations:** 1 Institute of Pharmacology, First Faculty of Medicine, Charles University and General University Hospital in Prague, Albertov 4, 128 00 Prague, Czech Republic; 2 Department of Psychiatry, First Faculty of Medicine, Charles University and General University Hospital in Prague, Ke Karlovu 11, 120 00 Prague, Czech Republic

**Keywords:** oxidative phosphorylation, mitochondrial respiration, reactive oxygen species, ATP, monoamine oxidase, antidepressants, agomelatine, ketamine, vortioxetine

## Abstract

This determination of the mitochondrial effect of pharmacologically different antidepressants (agomelatine, ketamine and vortioxetine) was evaluated and quantified in vitro in pig brain-isolated mitochondria. We measured the activity of mitochondrial complexes, citrate synthase, malate dehydrogenase and monoamine oxidase, and the mitochondrial respiratory rate. Total hydrogen peroxide production and ATP production were assayed. The most potent inhibitor of all mitochondrial complexes and complex I-linked respiration was vortioxetine. Agomelatine and ketamine inhibited only complex IV activity. None of the drugs affected complex II-linked respiration, citrate synthase or malate dehydrogenase activity. Hydrogen peroxide production was mildly increased by agomelatine, which might contribute to increased oxidative damage and adverse effects at high drug concentrations. Vortioxetine significantly reduced hydrogen peroxide concentrations, which might suggest antioxidant mechanism activation. All tested antidepressants were partial MAO-A inhibitors, which might contribute to their antidepressant effect. We observed vortioxetine-induced MAO-B inhibition, which might be linked to decreased hydrogen peroxide formation and contribute to its procognitive and neuroprotective effects. Mitochondrial dysfunction could be linked to the adverse effects of vortioxetine, as vortioxetine is the most potent inhibitor of mitochondrial complexes and complex I-linked respiration. Clarifying the molecular interaction between drugs and mitochondria is important to fully understand their mechanism of action and the connection between their mechanisms and their therapeutic and/or adverse effects.

## 1. Introduction

The primary role of mitochondria is as a source of energy in the form of adenosine triphosphate (ATP) for cellular processes. In addition, mitochondria are involved in calcium homeostasis, redox signaling, apoptosis regulation and heat production. Consequently, mitochondria have a key role in neurodevelopment and neuroplasticity. Thus, mitochondrial dysfunction and the consequent depletion of ATP production may play an important role in the pathophysiology of psychiatric disorders, including depression and may be a target for antidepressants. This is supported by many findings, including impaired mitochondrial membrane potential and damaged mitochondrial brain ultrastructure in a preclinical mouse model of chronic mild stress or reduced glucose utilization in certain brain areas of patients suffering from depression. Increased concentrations of oxygen and nitrogen species and lowered antioxidant protection lead to damage to nucleic acids, proteins and lipids. Increased markers of oxidative DNA damage, together with lowered DNA repair ability, have been found in patients with depression [1,2]. Based on these findings, the mitochondrial hypothesis has been postulated. It has been proven that mitochondria play a key role in neuroplasticity and neurodevelopment. The consequence of mitochondrial dysfunction is not only insufficient energy production but also impairment of neuronal communication, neuroinflammation, oxidative stress (as a result of redox imbalance) and reduced neuronal adaptation toward internal and external signals—neuroplasticity. Different studies have observed a stress-induced decrease in hippocampal neurogenesis and oxidative stress is a contributing factor. All these changes can participate in the development and progression of depression [1,3].

Currently, there is no single hypothesis covering all the signs and symptoms of depression, suggesting that depression pathophysiology has multiple mechanisms that are linked and lead to the same symptoms of the disease. The most currently discussed biological hypotheses potentially linked to mitochondrial dysfunction are the biogenic amine hypothesis, the genetic hypothesis, the environmental hypothesis, the immunological-inflammatory hypothesis, the abnormal glutamate receptor hypothesis and the neurotrophic hypothesis [4].

The current pharmacotherapy of depression relies especially on selective serotonin reuptake inhibitors (SSRIs) [5]. However, approximately 30% of patients treated with first-line SSRI treatment do not achieve full remission [6]. Many patients also suffer from SSRI side effects such as gastrointestinal irritation, sexual dysfunction, sleep disturbances and emotional blunting. [7,8]. Undoubtedly, there is a need to discover an antidepressant with a novel mechanism of action that would be efficacious for SSRI-unresponsive and/or SSRI-intolerant patients. Recently, the FDA/WHO approved antidepressants with different mechanisms of action, e.g., serotonin (5-HT) modulators and stimulators (vortioxetine), *N*-methyl-d-aspartate (NMDA) glutamate receptor antagonists (ketamine) and melatonin agonists and selective serotonin antagonists (agomelatine) [5,9].

Agomelatine (AGO) is a melatonergic antidepressant that is structurally similar to melatonin and affects circadian rhythms. It is a well-tolerated antidepressant, despite its hepatotoxicity. The most frequent adverse effects of AGO are headache, nasopharyngitis, back pain and upper respiratory tract infections [9,10,11].

Ketamine (KET), originally used as an anesthetic, has shown antidepressant effects at low subanesthetic doses. It is a potent antidepressant with a rapid onset that is effective in severe, drug-resistant and suicidal depression. Due to its complex mechanism of action and its effects, KET provides new insight into the pathophysiology of depression [12]. It has been suggested that the antidepressant effect of KET is mediated by energy metabolism and effects on antioxidant defense [13]. Moreover, it was shown that KET can increase BDNF expression and synthesis [14].

In general, low-dose and short-term KET administration is well tolerated; mild adverse effects include dizziness, vertigo, nausea, short-term dissociation and a blood pressure increase. High doses of KET administered for a prolonged time carry a risk of severe adverse effects, including acute anxiety, panic attack and a prolonged psychomimetic or dissociative effect [14,15,16,17].

Vortioxetine (VOR) is a multimodal antidepressant that acts as a serotonin transporter inhibitor and modulator of 5-HT receptors. The most common side effects were nausea and vomiting, dizziness, insomnia and sexual dysfunction. There are very little data available showing the effect of VOR on mitochondrial functions and cellular energy metabolism.

There is a growing body of evidence showing the relationship between mitochondrial dysfunction and depression. For example, patients with mitochondrial disorders are 3.9 times more prone to develop depressive comorbidity [18]. Muscle biopsy in patients with major depressive disorder (MDD) found significantly decreased mitochondrial ATP production rates, complex I activity and other enzyme ratios compared with healthy controls [18,19,20,21]. Many altered proteins identified in MDD are linked to oxidative phosphorylation (OXPHOS), e.g., the meta-analysis revealed decreased expression of complex I subunits in MDD and bipolar affective disorder. Our group reported impaired mitochondrial respiration in intact platelets from depressive patients, manifesting mostly as a decreased respiratory rate and maximal capacity of the electron transport chain (ETC). Similar findings were reported by Karabatsiakis et al. in peripheral blood mononuclear cells from patients with MDD; moreover, these results were negatively correlated with the severity of symptoms [22,23]. Interestingly, increased protein expression of complex I and ATP synthase was found in certain brain areas of depressive patients, which might be a compensatory mechanism for reduced energy supply [21]. It was previously shown that patients diagnosed with MDD have higher levels of 8-oxoguanine, a marker of oxidative DNA damage, pointing toward impaired mitochondrial function and oxidative imbalance [2]. Patients with MDD consistently show signs of oxidative DNA damage and increased lipid peroxidation compared to healthy controls, when both parameters improve with antidepressant treatment [24]. Peripheral blood mononuclear cell mitochondrial DNA from patients with MDD is more vulnerable to oxidative damage than DNA from controls [25]. It is believed that mitochondria might try to compensate for mtDNA damage by enhancing mitochondrial biogenesis.

In this study, we investigated the in vitro effects of three currently used antidepressants on mitochondrial energy metabolism and reactive oxygen species (ROS) production using isolated pig brain mitochondria as a biological model. Based on the mitochondrial dysfunction hypothesis, it can be assumed that the effects of some antidepressants can be targeted at mitochondrial dysfunction, primarily at the disruption of bioenergetics and oxidative stress.

## 2. Results

### 2.1. Activity of Mitochondrial Enzymes

**Figure 1:**
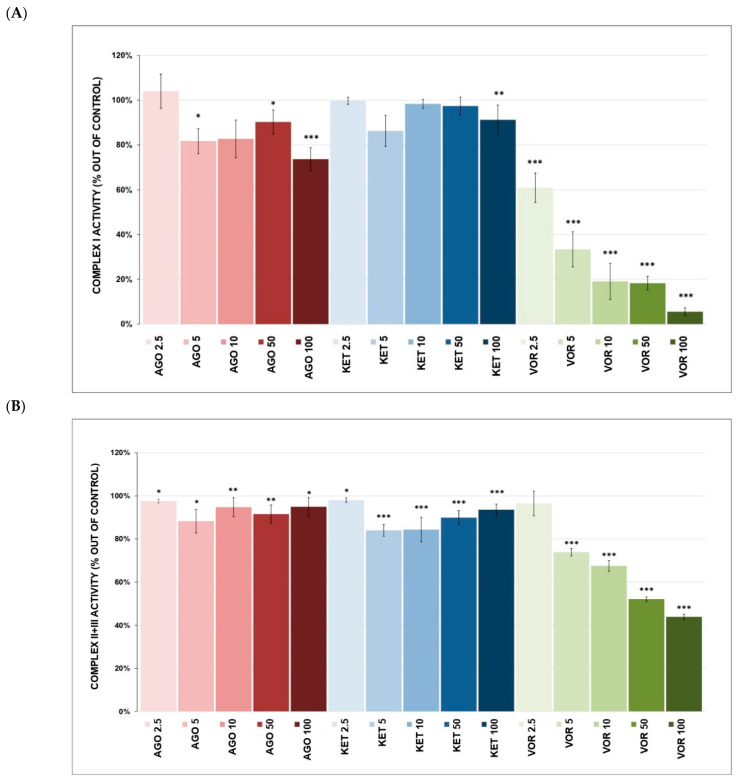

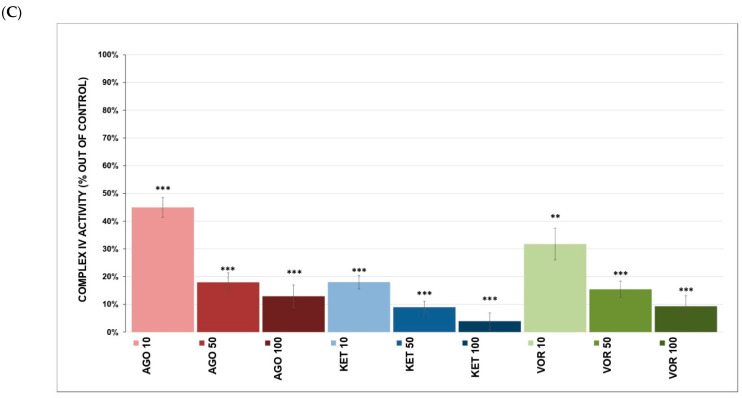
(**A**) Antidepressant-induced inhibition of complex I activity. Relative activity is displayed as 100% activity of control sample. The values are expressed as the mean ± SD for at least three independent measurements. Statistical significance was tested using a one sample *t*-test that mean value is equal to 100% and is expressed as * *p* ˂ 0.05. ** *p* ˂ 0.01. *** *p* ˂ 0.001. Drugs concentrations are expressed in µM. AGO—agomelatine, KET—ketamine, VOR—vortioxetine. (**B**) Antidepressant-induced inhibition of complex II+III activity. Relative activity is displayed as 100% activity of control sample. The values are expressed as the mean ± SD for at least three independent measurements. Statistical significance was tested using a one sample *t*-test that mean value is equal to 100% and is expressed as * *p* ˂ 0.05. ** *p* ˂ 0.01. *** *p* ˂ 0.001. AGO—agomelatine, KET—ketamine, VOR—vortioxetine. (**C**) Antidepressant-induced changes in complex IV activity. Relative activity is displayed as 100% activity of control sample. The values are expressed as the mean ± SD for at least three independent measurements. Statistical significance was tested using a one sample *t*-test that mean value is equal to 100% and is expressed as * *p* ˂ 0.05. ** *p* ˂ 0.01. *** *p* ˂ 0.001. Drugs concentrations are expressed in µM. AGO—agomelatine, KET—ketamine, VOR—vortioxetine.

The results of the mitochondrial ETC complexes activities are depicted in Figure 1A–C. VOR caused the dose-dependent and statistically significant inhibition of complex I (Figure 1A) at all concentrations (5.5 ± 1.8% at 100 μM, *p* < 0.001). VOR was also inhibited complex II+III activity (Figure 1B) (43.9 ± 1.2% at 100 μM, *p* < 0.001) and significantly inhibited complex IV activity (Figure 1C) (9.3 ± 3.8% at 100 μM, *p* < 0.001). 

KET-induced complex I inhibition (Figure 1A) reached statistical significance only at a concentration of 100 µM (91.2 ± 6.6%, *p* = 0.007). Nevertheless, KET inhibited complex II+III activity (Figure 1B) (93.6 ± 2.6% at 100 μM, *p* < 0.001) and complex IV activity (Figure 1C) (3.9 ± 3.0% at 100 μM, *p* < 0.001). 

AGO inhibited the activity of complex I (Figure 1A) (73.7 ± 5.1% at 100 µM, *p* < 0.001), complex II+III (Figure 1B) (94.9 ± 4.3 at 100 μM, *p* = 0.013) and also dose-dependently inhibited complex IV activity (Figure 1C) (45.0 ± 3.6% at 10 μM, *p* < 0.001; 17.9 ± 3.4% at 50 μM, *p* < 0.001; 12.8 ± 4.1% at 100 μM, *p* < 0.001).

Tested substances showed no or very little inhibitory/inductive properties toward CS and MDH (Table 1).

### 2.2. Mitochondrial Respiration

**Figure 2:**
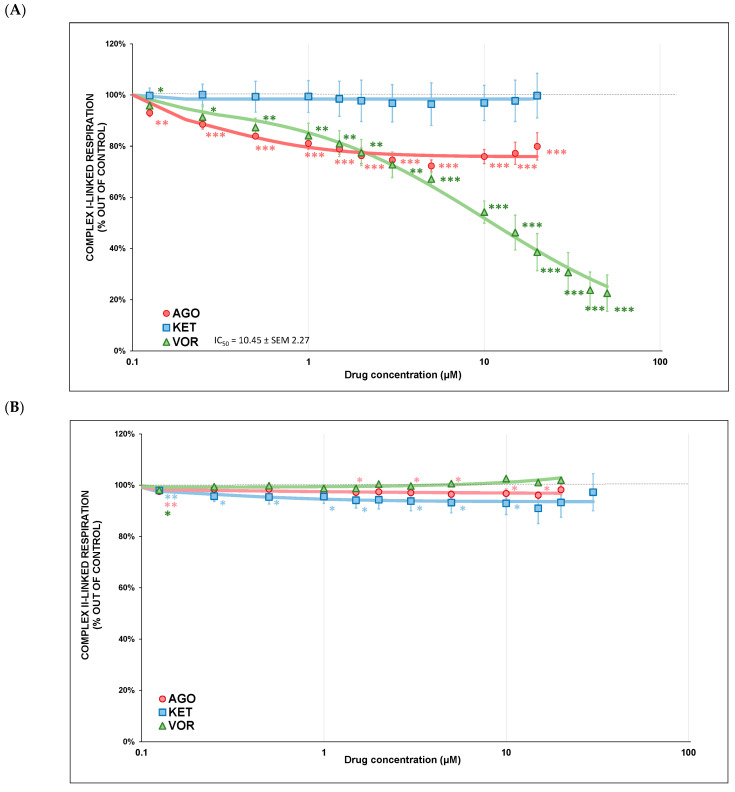
**(A**) Antidepressant-induced inhibition of complex I-linked respiration. Dose-response curves are displayed as plots of the respiration rate against drug concentration. Relative activity is displayed as 100% activity of control sample. Points are the mean of four independent measurements and lines represent the best/fitted curves using a four-parameter logistic function. Statistical significance was tested using a one sample *t*-test that mean value is equal to 100% and is expressed as * *p* ˂ 0.05. ** *p* ˂ 0.01. *** *p* ˂ 0.001. The half-maximal inhibitory concentration (IC_50_) was calculated for VOR. Drugs concentrations are expressed in μM. AGO—agomelatine, KET—ketamine, VOR—vortioxetine. (**B**) Antidepressant-induced inhibition of complex II-linked respiration. Dose-response curves are displayed as plots of the respiration rate against drug concentration. Relative activity is displayed as 100% activity of control sample. Points are the mean of four independent measurements and lines represent the best/fitted curves using a four-parameter logistic function. Statistical significance was tested using a one sample *t*-test that mean value is equal to 100% and is expressed as * *p* ˂ 0.05. ** *p* ˂ 0.01. *** *p* ˂ 0.001. Drugs concentrations are expressed in μM. AGO—agomelatine, KET—ketamine, VOR—vortioxetine.

Drug-induced changes in complex I-linked and complex II-linked respiration are depicted in Figure 2A,B. VOR was the only full inhibitor of complex I-linked respiration (Figure 2A) with a respiration rate of 22.5 ± 7.1 % (mean ± SD) at 50 μM (*p* < 0.001), IC_50_ = 10.5 ± 2.3 μM (mean ± SEM) and residual activity of 4.7 % ± 0.07 (mean ± SEM). KET inhibited complex I-linked respiration very weakly and AGO did not affect complex I-linked respiration at all (Figure 2A). In higher concentrations of all tested drugs, we observed the phenomenon of a rapid increase in the respiratory rate, which was probably caused by drug accumulation in mitochondrial membranes changing the lipid-protein interactions rather than by a direct drug-respiratory complex interaction [26,27,28,29]. Drug titration was terminated at the point of a sudden increase in the mitochondrial respiratory rate at high drug concentrations.

None of the three substances tested affected complex II-linked respiration (Figure 2B).

### 2.3. ATP Content and Kinetics

**Figure 3:**
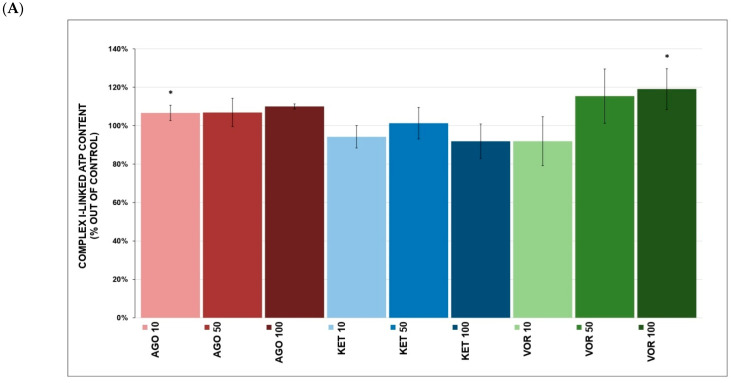

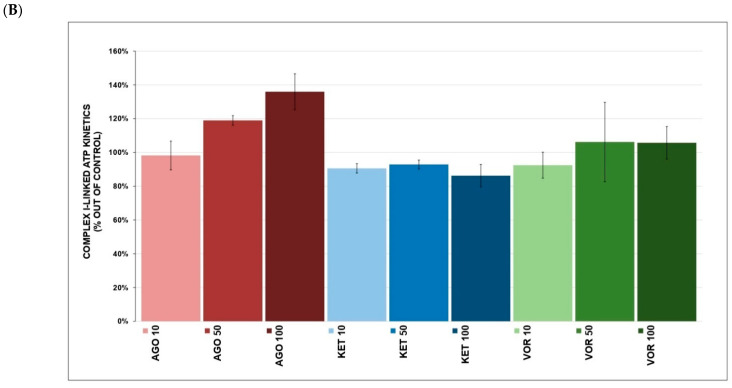
(**A**) Antidepressant-induced changes in complex I-linked ATP content. Relative activity is displayed as 100% activity of control sample (100% corresponded to a production of 160 nmol of ATP per 1 mg of protein). The values are expressed as the mean ± SD for at least six independent measurements. Statistical significance was tested using one sample *t*-test that mean value is equal to 100% and is expressed as * *p* ˂ 0.05. Drugs concentrations are expressed in µM. AGO—agomelatine, KET—ketamine, VOR—vortioxetine. (**B**) Antidepressant-induced changes in complex I-linked ATP kinetics. Relative activity is displayed as 100% activity of control sample (100% corresponded to a production of 282 nmol of ATP per 1 mg of protein per 1 min). The values are expressed as the mean ± SD for at least six independent measurements. Statistical significance was tested using one sample *t*-test that mean value is equal to 100%. Drugs concentrations are expressed in µM. AGO—agomelatine, KET—ketamine, VOR—vortioxetine.

**Figure 4:**
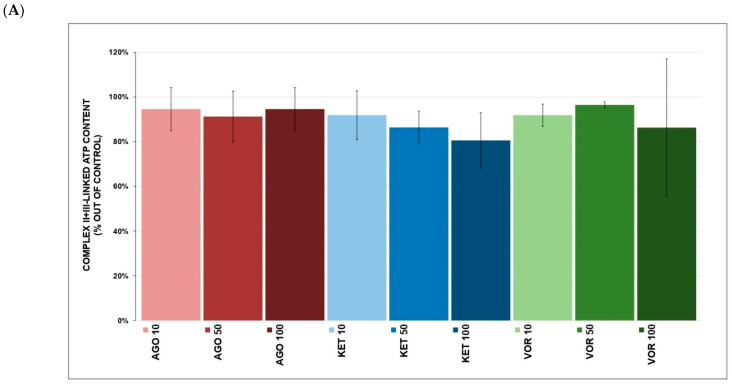

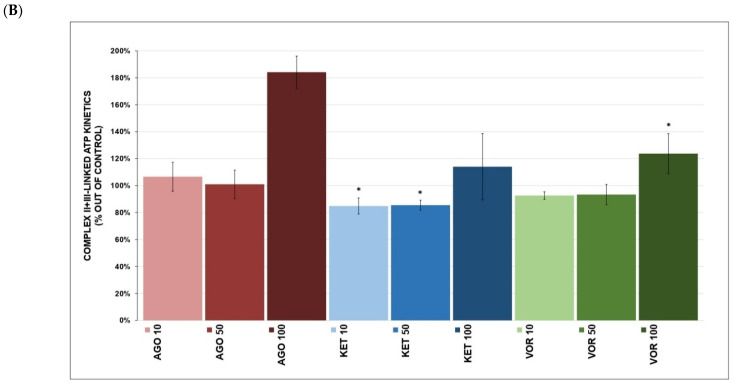
(**A**) Antidepressant-induced changes in complex II-linked ATP content. Relative activity is displayed as 100% activity of control sample (100% corresponded to a production of 291 nmol of ATP per 1 mg of protein). The values are expressed as the mean ± SD for at least six independent measurements. Statistical significance was tested using one sample *t*-test that mean value is equal to 100%. Drugs concentrations are expressed in µM. AGO—agomelatine, KET—ketamine, VOR—vortioxetine. (**B**) Antidepressant-induced changes in complex II-linked ATP kinetics. Relative activity is displayed as 100% activity of control sample (100% corresponded to a production of 1289 nmol of ATP per 1 mg of protein per minute). The values are expressed as the mean ± SD for at least six independent measurements. Statistical significance was tested using one sample *t*-test that mean value is equal to 100% and is expressed as * *p* ˂ 0.05. Drugs concentrations are expressed in µM. AGO—agomelatine, KET—ketamine, VOR—vortioxetine.

The ATP content and kinetics results are depicted in Figure 3A,B and Figure 4A,B, respectively.

Complex I-linked ATP content (Figure 3A) was increased after VOR (119.0 ± 10.6% at 100 μM, *p* = 0.037, mean ± SD) and AGO (106.4 ± 4.0% at 10 μM, *p* = 0.044), but KET had no significant effect. Complex I-linked ATP kinetics (Figure 3B) were not affected by any tested drug. None of the tested drugs significantly affected complex II+III-linked ATP content (Figure 4A). Complex II+III-linked ATP kinetics (Figure 4B) was significantly affected by KET (84.9 ± 5.9% at 10 μM, *p* = 0.048) and VOR (92.6 ± 2.8% at 10 μM, *p* = 0.046).

### 2.4. Hydrogen Peroxide Production

**Figure 5:**
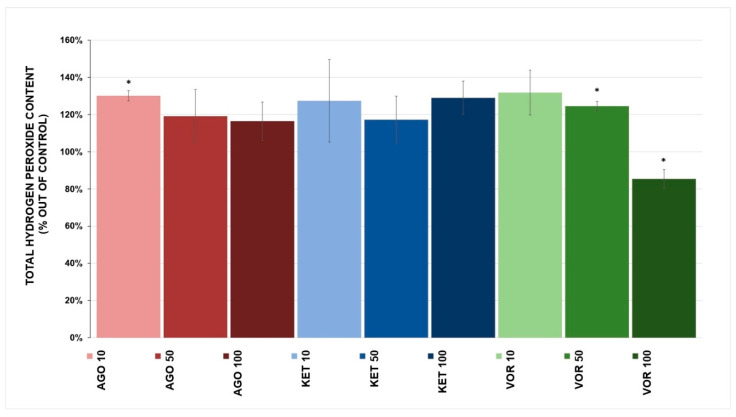
Antidepressant-induced changes in total hydrogen peroxide (H_2_O_2_) content. Relative activity is displayed as 100% activity of control sample (100% corresponded to a production of 450 pmol of H_2_O_2_ per 1 mg of protein). The values are expressed as the mean ± SD for at least three independent measurements. Statistical significance was tested using a one sample t-test that mean value is equal to 100% and is expressed as * *p* ˂ 0.05. Drugs concentrations are expressed in μM. AGO—agomelatine, KET—ketamine, VOR—vortioxetine.

Changes in H_2_O_2_ production were observed in all tested drugs, total H_2_O_2_ content is depicted in Figure 5. The statistically significant increase in H_2_O_2_ content was caused by AGO (130.1 ± 2.7% at 10 μM, *p* = 0.041) and by VOR (124.5 ± 2.5% at 50 μM, *p* = 0.046). However, VOR at 100 μM decreased the total H_2_O_2_ content (85.4 ± 5.0%, *p* = 0.037). KET showed no statistically significant effect on total H_2_O_2_ production.

### 2.5. MAO Activity

**Figure 6:**
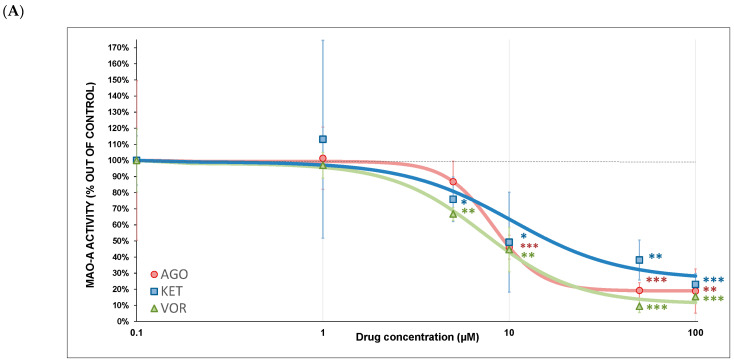

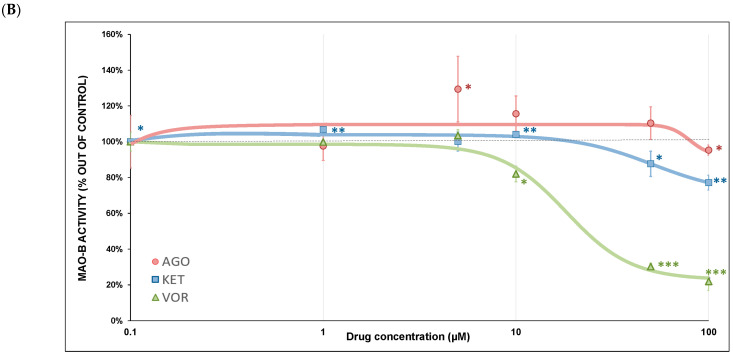
(**A**) Antidepressant-induced MAO-A inhibition. The relative activity is displayed as 100% activity of the control sample. Statistical significance was tested using a one sample *t*-test that mean value is equal to 100% and is expressed as * *p* ˂ 0.05. ** *p* ˂ 0.01. *** *p* ˂ 0.001. The half-maximal inhibitory concentration (IC_50_), Hill slope and residual activity was calculated (Table 2). Drugs concentrations are expressed in μM. AGO—agomelatine, KET—ketamine, VOR—vortioxetine. (**B**) Antidepressant-induced MAO-B inhibition. The relative activity is displayed as 100% activity of the control sample. Statistical significance was tested using a one sample *t*-test that mean value is equal to 100% and is expressed as * *p* ˂ 0.05. ** *p* ˂ 0.01. *** *p* ˂ 0.001. The half-maximal inhibitory concentration (IC_50_), Hill slope and residual activity was calculated (Table 2). Drugs concentrations are expressed in μM. AGO—agomelatine, KET—ketamine, VOR—vortioxetine.

All three tested substances were found to be partial inhibitors of MAO-A (Figure 6A). The strongest MAO-A inhibitor was VOR (IC_50_ = 7.33 ± 1.1 µM), followed by AGO (IC_50_ = 8.20 ± 1.4 µM) and KET (IC_50_ = 10.36 ± 8.3 µM). MAO-B activity (Figure 6B) was partially inhibited by VOR (IC_50_ = 18.24 ± 3.5 µM); KET showed only a weak MAO-B inhibition (IC_50_ = 51.16 ± 25.5 µM); AGO did not affect its activity at all (kinetic parameters were not calculated). Drug-induced MAO-A and MAO-B inhibition curves are depicted in Figure 6A,B and the kinetic parameters are summarized in Table 2.

### 2.6. Correlations

Statistically significant correlations were identified between measured mitochondrial parameters using the Pearson correlation coefficient. Strong and statistically significant correlations were found between the activity of complex I and the activity of complex II+III, between the activity of complex II+III and the activity of complex IV, between the activity of complex II+III and total ROS content, between the activity of complex II+III and complex I-linked respiration, and between the complex II-linked ATP kinetics and complex I-linked respiration for VOR. For AGO, there were found to be significant correlations between the activities of complex IV and complex I-linked ATP kinetics and between complex I-linked ATP kinetics and complex II-linked ATP kinetics. The complete results of the correlation analysis are summarized in Table 3 and Table 4.

## 3. Discussion

### 3.1. Mitochondrial Enzyme Activity and Respiration

All three tested drugs significantly inhibited the activity of mitochondrial complexes I, II+III and IV (Figure 1A–C); complex IV was the most affected. VOR was the most potent inhibitor of individual ETC complexes; complex I was inhibited to 5.5% (at 100 μM) and complex I-linked respiration (Figure 2A) was fully inhibited by VOR (IC_50_ = 10.5 μM). Complex I is usually the most vulnerable part of OXPHOS, and there are more than 60 well-known inhibitors of complex I, especially lipophilic molecules [30]. Complex I is also a potent ROS producer; its functional impairment can lead to both insufficient ATP production and increased oxidative damage. VOR also significantly decreased the activity of complexes II+III and IV (Figure 1B,C). Because complex II+III is an alternative electron input to OXPHOS, this inhibitory effect on mitochondrial respiratory complexes could lead to OXPHOS impairment and insufficient energy supply. Although VOR administration lowered the activity of all individual complexes, it was no surprise that it also acted as a potent inhibitor of mitochondrial complex I-linked respiration (Figure 2A). However, complex II-linked respiration (Figure 2B) remained unaffected by VOR, suggesting that OXPHOS is still able to perform respiration through complex II even at very high VOR concentrations. Borhannejad et al. found no significant difference in adverse events between a group of patients taking VOR and a group of patients taking sertraline. Since the sample size of this study was rather small (40 patients in total), the clinical relevance of these results may be questionable. Rare adverse reactions may also not be visible with this small sample size [31]. Another study shared similar results comparing drug-induced adverse effects and therapy discontinuations in patients taking escitalopram, desvenlafaxine and VOR [32]. Overall, VOR is considered to be safe and tolerable. Meta-analyses showed higher rates of treatment discontinuation in the VOR group than in the placebo group but lower rates than in the active control group. It was reported that its tolerability worsens with increasing doses [33,34,35]. However, we can speculate that its inhibitory effect on ETC complexes and mitochondrial respiratory rate might play a part in the adverse effects of high VOR concentrations.

These results are in accordance with our previous data for tricyclic antidepressants and SSRIs, which extensively inhibited mitochondrial respiration and the activity of respiratory chain complexes at high drug concentrations when the most affected complexes were complexes I and IV [36]. It can be assumed that this conformity in antidepressant-induced mitochondrial dysfunction could be a consequence of similar drug-induced changes in mitochondrial functions. In the search for new biological markers of treatment response, it was found that higher basal complex I and CS activity and a higher treatment-induced complex I activity decrease were directly linked to better response to SSRI treatment in patients with MDD [20].

KET and AGO had almost no effect on the activity of complexes I, II+III (Figure 1A,B) and the mitochondrial respiratory rate (Figure 2A,B). However, they were very potent inhibitors of complex IV (Figure 1C). Complex IV is the rate-limiting player in OXPHOS, initiating the final step in the ETC and its activity is also coupled with neuronal activation. It was previously reported that antidepressants might have both stimulating and inhibiting properties toward complex IV activity, while other psychoactive drugs mostly tend to increase their activity [37]. The current results for KET are in accordance with our previous results as follows: KET is only a very weak inhibitor of complex I-linked respiration (Figure 2A) and shows no inhibitory activity against complex II-linked respiration (Figure 2B) [30]. Other studies describing KET-induced mitochondrial changes reported the following several inconsistent results: reversed chronic mild stress-induced inhibition of complexes I, II and IV in rats; increased complex IV activity in rats after subchronic KET administration; no significant effect on mouse brain and macrophage mitochondria [38,39,40].

Our results for AGO are in accordance with Kumar et al., who showed impaired activity of mitochondrial complexes after subchronic AGO administration to rats as follows: complex I activity was increased mostly by a 10 mg/kg dose, whether higher doses decreased complex I activity; complex II activity was increased by the highest dose of 50 mg/kg; complex IV activity was decreased by lower doses and increased by the highest dose [41]. In in vitro studies, AGO was able to normalize the impaired activity of mitochondrial complexes and ROS increase in rats induced by prenatal exposure to valproic acid and reverse galactose-induced mitochondrial dysfunction [42,43]. The inconsistency of our results compared to these studies is most likely the result of different methodologies (in vivo vs. in vitro). We observed a direct molecular effect of antidepressants on mitochondrial parameters without the involvement of other biological pathways or an effect on the whole organism.

### 3.2. ATP Production

We separately studied complex I- and complex II-linked ATP content and kinetics to better understand the molecular mechanisms of drug action. VOR significantly increased complex I-linked ATP content (Figure 3A) and complex II-linked ATP kinetics (Figure 4A). This was quite surprising that VOR showed the most potent inhibitory properties toward individual ETC complexes and complex I-linked respiration. We hypothesize that due to this inhibition, some adaptation mechanisms could be triggered to preserve ATP formation through complex II-linked respiration. There are no comparative data describing the effect of VOR on ATP kinetics.

Complex II-linked ATP kinetics (Figure 4B) were significantly decreased by KET, which might be linked to the strongest KET-induced inhibition of complex IV as a rate-limiting process in ATP formation. It was previously reported that KET treatment reduced ATP/ADP metabolic ratios in rodents, which correlated with a forced-swim test time. It was also suggested that KET might cause energetic deficits by stimulating anabolic processes that consume ATP and require higher ATP production, which could lead to OXPHOS stimulation and ROS increases [13]. Moreover, KET-treated pluripotent stem cell-derived neurons produced less ATP than untreated controls [44].

AGO-mediated insignificant stimulation of both complex I- and complex II-linked ATP kinetics (Figure 3B and Figure 4B) was also observed. We suggest that higher inhibitory complex I activity and complex I-linked respiration (compared to KET) lead to potential adaptive mechanisms, similar to VOR.

All these findings suggest that mitochondrial ATP production is a very complex process that cannot be simply derived from the activity of isolated ETC complexes or the oxygen consumption rate.

### 3.3. Hydrogen Peroxide Production

We did not observe a trend in increased H_2_O_2_ production by mitochondria (Figure 5) with increasing concentrations of the tested antidepressants. The highest significant H_2_O_2_ content increase was caused by AGO at a concentration of 10 µM. In a rodent study, subchronic AGO administration did not significantly affect oxidative stress markers, but there was a small effect on antioxidant systems [41]. AGO normalized galactose-induced ROS increase in a rat model of hippocampal aging and reduced oxidative stress and damage in vitro in PC12 cells [43,45].

VOR, similar to AGO, stimulates H_2_O_2_ production at lower concentrations. At a concentration of 100 µM, VOR was able to decrease total H_2_O_2_ production, which might be linked to the inhibition of complex I activity as the most potent producer of ROS.

The effect of KET on mitochondrial H_2_O_2_ production was insignificant, showing a mild stimulation of its production. In stem cell-derived neurons, KET treatment significantly increased ROS production, which might not be directly linked to mitochondrial ROS [44].

In addition to cellular oxidative damage, ROS play important signaling functions, including activating guanylate cyclase and are also essential for long-term neuronal plasticity by modulating the activities of several kinases and phosphatases. The peroxide anion reacts with nitric oxide, creating peroxinitrite, a compound that disrupts the enzymatic function of tyrosine residues, which could decrease monoamine neurotransmitter production, worsening depressive symptoms.

Direct binding of antidepressants to ETC complexes might lead to an increase in ROS production, as was seen with cannabinoid drugs [46]. However, there are also data that indicate that antidepressants, regardless of their mechanism of action, can improve oxidative stress parameters in a subpopulation of patients, thus showing a capacity to improve antioxidant mechanisms. An increased level of oxidative stress was also found to be a marker of a poorer response to SSRI treatment [20]. Because ROS are also important signaling molecules, it is difficult to determine the consequences of antidepressant-induced changes in ROS levels. We observed a drug-induced increase in total ROS production of approximately 30%, which is not an increase of approximately hundreds of percent that would reliably indicate an increase that would lead to increased oxidative damage. This mild an ROS increase could lead to signaling changes, and this hypothesis should be further tested. There is a possibility that ROS increase might participate in adverse effects associated with treatment. More studies observing lipid peroxidation, mtDNA damage and other markers of increased oxidative stress are needed to understand whether this ROS increase is on the “physiological” or the “pathophysiological” side.

### 3.4. MAO Inhibition

All tested drugs were partial inhibitors of monoamine oxidase A (Figure 6A) (MAO-A). The MAO-A isoform is an important metabolic enzyme of serotonin, norepinephrine and dopamine, which are neurotransmitters closely linked to depression. This mechanism could participate in their antidepressive effect, and MAO inhibitors were used as the first antidepressants [47].

MAO-B activity was significantly inhibited by VOR (Figure 6B). MAO-B is preferentially responsible for dopamine, benzylamine, phenethylamine and tyramine oxidative deamination. MAO-B inhibition is therapeutically used for the treatment of neurodegenerative disorders, e.g., Alzheimer’s or Parkinson’s disease, whereby the reduced production of H_2_O_2_ during MAO-catalyzed oxidation of monoamine neurotransmitters may be decisive [48]. In three large placebo-controlled studies, VOR significantly improved memory impairment in patients with MDD. Our results indicate that VOR-induced MAO-B inhibition may participate in the VOR-induced decrease in H_2_O_2_ production and might be one of the mechanisms by which VOR improves cognitive deficits [49].

### 3.5. Correlations

Strong and significant positive correlations were found between complex I and complex II+III activity and between complex II+III and complex IV activity for VOR. The correlation between individual complexes is logical; both complex II+III and complex IV are the “next steps” in the ETC and should be affected by previous complexes. Another strong correlation was found between the activity of complex II+III and the total ROS content. ROS production should be linked to individual ETC complex activity, and this finding suggests a causal correlation between these two parameters. A strong correlation was also found between complex II+III activity and complex I-linked respiration, indicating that the activity of complex II+III might also affect complex I-linked mitochondrial respiration, but this is a highly complex process that is not strictly dependent only on ETC complex activities.

The correlations between complex II-linked ATP kinetics and complex I-linked respiration (VOR) and between complex II-linked ATP kinetics and complex IV activity/complex I-linked ATP kinetics (AGO) might involve the previously mentioned activation of adaptation mechanisms but also might not be causal. Further research observing the connection between these parameters is needed.

### 3.6. Study Limitations

We investigated the effect of currently used antidepressants (AGO, KET and VOR) on mitochondrial parameters in the purified mitochondrial fraction, which allows more accurate recognition of drug effects on compensatory and regulatory mechanisms in mitochondria than in in vivo measurements. However, it must be noticed, that the investigation of the regulatory and compensatory brain mechanisms and other pathways that can influence the effects of the tested drugs on mitochondrial functions in vivo is beyond the experimental approach of this study.

We used a wide range of drug concentrations (units to tens of µM) in our measurements to observe their effect on mitochondrial functions, some of which were much higher than therapeutic plasma concentrations in vivo (tenths to units of µM). All tested antidepressants are lipophilic molecules with a high probability of accumulation in neuronal membranes and subcellular structures [50,51,52,53,54,55]. A high antidepressant concentration might be expected in the mitochondria due to several mechanisms of xenobiotic accumulation [56]. High drug concentrations allowed us to determine the correlation between individual parameters at a concentration achievable in the brain at overdose, where significant drug-induced mitochondrial dysfunction occurred. Pig brain mitochondria were used as a biological model and were established and evaluated previously as a suitable model for studies investigating mitochondrial drug effects [57]. Purified mitochondria allow for studying direct drug-induced changes in mitochondrial parameters using suitable substrates, inhibitors and uncouplers. The further transition of this research to cell cultures is expected, especially for drugs that showed significant effects in therapeutic concentrations.

### 3.7. Possible Clinical Impact

The results of this study showed that AGO, KET and VOR significantly affected mitochondrial parameters in different ways, which indirectly supports the neurotrophic hypothesis of depression. Decreased levels of neurotrophic factors such as BDNF could directly affect mitochondrial performance because they act as mitochondrial respiratory couplers on complex I, which enhance mitochondrial energy production. Both a lack of BDNF and decreased mitochondrial performance contribute to disturbances in neuroplasticity and neurodevelopment. However, there is clear evidence of a connection between mitochondrial and neurotrophic hypotheses at this level [58].

It can be hypothesized that long-term drug-induced inhibition of individual respiratory complexes (especially complex IV, in this case) may cause mitochondrial toxicity manifesting as adverse drug effects [59]. In addition to lowered ATP formation and increased ROS production, mitochondrial dysfunction could activate immune and inflammatory processes, which likely contribute to adverse drug effects. Mitochondria can release oxidized mitochondrial DNA, and other factors act as proinflammatory mediators. This could serve as evidence connecting the mitochondrial and immune-inflammatory hypotheses [58].

Mitochondrial functions and cell energy metabolism are tightly linked with the pathophysiology of psychiatric diseases; therefore, measuring the mitochondrial functions of patients and considering the bioenergetic profile of individual psychiatric diseases should be involved in clinical practice in advance to choose the most appropriate medication. This is relevant, especially in patients who do not respond to treatment, are pharmacoresistant, or have an unidentified mitochondrial disease. Identifying potential modulators of treatment response (e.g., mitochondrial dysfunction) could help to optimize and personalize the pharmacological treatment of psychiatric diseases [60].

## 4. Materials and Methods

All materials and methods have been described in our previous article; only brief summary follows [59]. New protocol for ATP kinetics and content was established.

### 4.1. Media and Chemicals

The composition of the media used was the following: mitochondrial isolation medium: 0.32M sucrose and 4 mM HEPES (pH 7.4); respiratory medium (MiR05 without BSA):110 mM sucrose, 60 mM K^+^-lactobionate, 20 mM taurine, 3 mM MgCl_2_·6H_2_O, 10 mM KH_2_PO_4_, 0.5 mM EGTA and 20 mM HEPES (7.1); Krebs-Henseleit (KH) buffer: 118 mM NaCl, 4.7 mM KCl, 1.2 mM KH_2_PO_4_, 25 mM NaHCO_3_ and 11.1 mM glucose. Chemicals were purchased from Sigma-Aldrich Co. (St. Louis, MO, USA). The 5-Hydroxytryptamine [^3^H] trifluoroacetate ([^3^H]serotonin) and 2-phenylethylamine [ethyl-1-^14^C] hydrochloride ([^14^C]PEA) were purchased from American Radiolabeled Chemicals, Inc. (St. Louis, MO, USA). AGO, KET and VOR were dissolved in DMSO in corresponding concentrations.

### 4.2. Isolation of Pig Brain Mitochondria

Pig brains were obtained from a slaughterhouse. Mitochondrial fraction isolation and purification from brain cortex were performed as previously described [59]. Briefly, grey matter from pig brains was homogenized and crude mitochondrial fraction was isolated and then centrifuged on the sucrose gradient to obtain purified mitochondrial fraction. The freshly purified mitochondria were kept on ice until the assays were performed and were used for measurements of the mitochondrial oxygen consumption rate, measurements of ATP and ROS formation. Frozen mitochondria (stored at −70 °C) were used for the following enzyme activity measurements: ETC complexes, citrate synthase (CS) and malate dehydrogenase (MDH) activity.

### 4.3. Activities of Mitochondrial Enzymes

Ultrasonication and incubation of mitochondria with the tested drugs for 30 min at 30 °C was performed before measurement with a corresponding drug-free control (DMSO) for every measurement. Mitochondrial enzymes activities were determined spectrophotometrically as absorbance using a GENESYS 180 UV-Vis Spectrophotometer (Thermo Fisher Scientific, Waltham, MA, USA).

### 4.4. Citrate Synthase Activity

The reaction mixture consisted of Triton, Tris, 5,5′-dithiobis-(2-nitrobenzoic) acid and acetyl coenzyme A. The reaction was started by adding oxaloacetate and the CS activity was measured by detecting the color change of 5,5′-dithiobis-(2-nitrobenzoic) acid at 412 nm with a duration of 3 min. The final protein concentration was 20 µg/mL and final drug concentration 100 µM. Each measurement had a corresponding drug-free control (DMSO).

### 4.5. Malate Dehydrogenase Activity

The conversion of oxaloacetate to malate was used to measure the activity, and it was measured at 340 nm for 3 min. The reaction was initiated by adding oxaloacetate and nicotinamide adenine dinucleotide (NADH). The final protein concentration was 20 µg/mL and final drug concentration 100 µM. Each measurement had a corresponding drug-free control (DMSO).

### 4.6. Complex I (NADH Dehydrogenase) Activity

The reaction mixture consisted of KH_2_PO_4_, MgCl_2_ and KCN and the reaction was started by adding decylubiquinone and NADH. The rotenone-sensitive NADH oxidation reaction was measured at 340 nm for 5 min. The final protein concentration was 150 µg/mL and final drugs concentrations 2.5, 5, 10, 50 and 100 µM. Drug-free control (DMSO) was used as a control for each measurement.

### 4.7. Complex II+III (Succinate Cytochrome c Oxidoreductase) Activity

The activity of complex II+III was measured with an antimycin A-sensitive cyt *c* reduction at 550 nm for 3 min, and it was initiated by the addition of cyt *c*. The medium consisted of KH_2_PO_4_, EDTA, KCN and rotenone. The final protein concentration was 50 µg/mL and final drugs concentrations 2.5, 5, 10, 50 and 100 µM. Drug-free control (DMSO) was used as a control for each measurement.

### 4.8. Complex IV (Cytochrome c Oxidase) Activity

The medium consisted of KH_2_PO_4,_ and the reaction was initiated by reduced cyt *c*. The decrease in absorbance was measured at 550 nm for 3 min. The final protein concentration was 10 µg/mL and final drugs concentrations 10, 50 and 100 µM. Drug-free control (DMSO) was used as a control for each measurement.

### 4.9. ATP Content and Kinetics

The following protocol has been modified and optimized [61,62,63,64]. An ATP Bioluminescence Assay Kit CLS II was used to measure ATP content and kinetics. The luminescence was measured using FluoroMax-3 (Jobin Yvon, Edison, NJ, USA) at 562 nm. Standard curve for ATP determination was prepared from ATP standard in the range from 0 to 600 nM. Tested substances were used at final concentrations 10, 50 and 100 µM, with drug-free control (DMSO) for each measurement and the final protein concentration was 50 µg/mL.

### 4.10. Total Complex I- and Complex II+III-Linked ATP Content

Mitochondria were incubated for 30 min on ice with the MiR05 buffer, tested substances and mitochondria. Consequently, the substrate mix was added, consisting of 5 mM malate and 5 mM pyruvate (for complex I) or 5 mM succinate and 1 μM rotenone (for complex II), 60 μM ADP and 0.75 mM MgCl_2_·6H_2_O and incubated for 30 min in 30 °C. Reaction was stopped by heating vials at 100 °C for 2 min. Background luminescence was measured. In total, 230 µL of luciferase reagent was added and total ATP° content for complex I and complex II+III was determined by measuring the luminescence for 1 min. Drug-free control (DMSO) was part of each measurement.

### 4.11. Complex I- and Complex II+III-Linked ATP Kinetics

For ATP kinetics determination MiR05 buffer, mitochondria and drugs were incubated on ice for 20 min and another 10 min at room temperature. Background luminescence was measured. Consequently, the same substrate mixture as mentioned above, and 230 µL of luciferase reagent were added and ATP kinetics was determined by measuring the luminescence for 4 min. Each measurement had a corresponding drug-free control (DMSO). Previous study confirmed high correlation between the rate of polarographic- and bioluminescence-derived ATP production. Thus, further agreement between polarographic-based and luciferase-based measurements of ATP production was not verified in this study [63].

### 4.12. Hydrogen Peroxide Production

Hydrogen peroxide formation was determined using an Amplex Red Hydrogen Peroxide/Peroxidase Assay Kit. The reaction mixture consisted of 27 mM HEPES (pH 7.4), 114 mM sucrose, 100 mM KCl, 1.3 mM K_2_HPO_4_, 5 mM malate, 5 mM pyruvate, 1 µM rotenone, 10 mM succinate, 0.75 mM MgCl_2_, 50 mM ADP, 15 µM Amplex Red and horseradish peroxidase 0.09 U/ML. Mitochondria were incubated with the tested substances for 30 min at 30 °C at final concentrations 10, 50 and 100 µM and each measurement had a corresponding drug-free (DMSO) control. Fluorescence of Amplex Red was observed at an excitation of 571 nm and an emission of 585 nm for 1 min. The reaction was stopped by the addition of antimycin A. The standard curve for hydrogen peroxide was prepared using H_2_O_2_ standard.

### 4.13. Mitochondrial Respiration

The mitochondrial oxygen consumption rate was measured by high-resolution respirometry using Oxygraph-2k (Oroboros Instruments Corp, Innsbruck, Austria). The final protein concentration was 0.05–0.14 mg/mL and the reaction mixture consisted of 2 mM malate, 5 mM pyruvate, 1.25 mM ADP and 0.75 M MgCl_2_ for complex I-linked respiration; 1.25 mM ADP, 0.75 mM MgCl_2_, 1 µM rotenone and 10 mM succinate for complex II-linked respiration. The following four simultaneous measurements were assessed: a titration with the drug to the final drug concentrations of 0.125–100 µM in one chamber and a titration with a drug-free control (DMSO) in the second oxygraph chamber.

### 4.14. Monoamine Oxidase Activity

Mitochondria in KH buffer at final concentration of 800 µg/mL were preincubated with the tested drugs in a final concentration range of 0.1–300 µM for 60 min at 37 °C. For each measurement, there was a corresponding drug-free control (DMSO). Radiolabeled substrates ([^3^H]serotonin for MAO-A and [^14^C]PEA for MAO-B) were added to initiate the reaction. The reaction was carried out at 37 °C for 20 min for MAO-A and for 1 min for MAO-B, and the reaction was stopped by hydrochloric acid. The radioactivity of the organic phases of the extracts were measured by liquid scintillation counting (LS 6000IC, Beckman Instruments, Inc., Fullerton, CA, USA) [65].

### 4.15. Data Analysis and Statistics

The mitochondrial enzyme activities and ATP kinetics data were measured and calculated as the slope of the time dependence of absorbance or fluorescence. ATP and ROS contents were measured, and the means of the time-dependent fluorescence curves were calculated. The control sample activity was 100%, and the drug effect was expressed as a % of the control. ATP and H_2_O_2_ standard curves were constructed.

DatLab 7.4 software (Oroboros Instruments, Innsbruck, Austria) was used for high-resolution respirometry data collection and analysis it displays oxygen flux and the real-time oxygen concentration. The respiration rate was expressed as pmol O_2_ consumed/second/mg of a protein.

The inhibition of respiration rate and MAO activity were analyzed by A four-parameter logistic regression with Prism software (GraphPad Software, San Diego, CA, USA) was used to analyze the respiratory rate inhibition and activity of MAO. These data were used to establish the half-maximal inhibitory concentration (IC_50_), residual activity and the Hill slope. The IC_50_ represents the drug concentration required to inhibit the difference between the baseline and the residual value of the mitochondrial oxygen flux or MAO activity by 50%.

Data analysis was performed with STATISTICA 12 analysis software (TIBCO Software Inc., Palo Alto, CA, USA) using one-sample *t* test. Data are expressed as the mean ± standard deviation (SD) or the mean ± standard error of the mean (SEM). The correlation matrix and Pearson correlation coefficient were used to identify and display statistically significant correlations between individual parameters. Respiratory parameters, which were not affected by drugs, were excluded from the correlation analysis (for this reason all KET data were excluded).

## 5. Conclusions

Our in vitro study with currently used antidepressants revealed important and statistically significant drug-induced changes in OXPHOS. All three tested antidepressants decreased the activity of ETC complexes at higher concentrations. Reduced activity of ETC complexes should cause reduced ATP generation in the OXPHOS system. All three antidepressants were very potent inhibitors of complex IV activity, which is the rate-limiting complex for ATP generation. It can be speculated that the drug-induced maintenance or increase in ATP kinetics might be an adaptation mechanism to address insufficient respiration through complex I or by switching primarily to complex II-linked respiration, which remained preserved despite the drug-induced inhibition of complex II+III. Likely, long-term inhibition of OXPHOS could be linked to a neuronal ATP deficit, which could negatively contribute to neuronal damage at very high concentrations of the drug.

Considering the overall drug-induced changes in mitochondrial parameters in relation to adverse effects, long-term inhibition of complex IV likely compromises physiological processes and could be involved in adverse drug reactions. The most pronounced drug-induced mitochondrial dysfunction-related adverse effect could be linked to VOR as the most potent inhibitor of individual ETC complexes and complex I-linked respiration.

We also noticed an effect on ROS production. The VOR-induced decrease in H_2_O_2_ concentrations suggests the activation of antioxidant mechanisms. Drug-induced changes in H_2_O_2_ concentration affect redox balance and signaling cascades. Further research is needed to describe whether drugs increase oxidative damage (which is involved in undesirable effects) or promote the activation of antioxidant defense.

Monoamine oxidase inhibition likely plays a part in the desirable effects of these substances. All three tested antidepressants acted as partial MAO-A inhibitors, suggesting additional antidepressive effects in addition to their primary mechanism of action. VOR also partially inhibited MAO-B, which might be linked to its ability to decrease total H_2_O_2_ content, lower oxidative damage and improve cognitive deficits in MDD patients. In vitro studies have limitations and further cell culture, and in vivo research is needed to clarify the connections among the pathophysiology of MDD, mitochondrial activity and the mitochondrial effect of psychoactive drugs.

## Figures and Tables

**Table 1:** The effect of antidepressants on citrate synthase (CS) and malate dehydrogenase (MDH) activity.

Drug	Concentration (μM)	CS (% of Control)			N	MDH (% of Control)			N
agomelatine	10 100	** 108.8 ** 95.7	± ±	1.06 0.55	3 3	101.7 101.0	± ±	1.99 2.31	3 3
ketamine	10	107.3	±	3.91	3	97.4	±	2.98	3
	100	97.4	±	1.08	3	101.7	±	2.61	3
vortioxetine	10	109.3	±	6.11	3	106.9	±	6.80	3
	100	* 96.9	±	1.08	3	100.2	±	1.82	3

The values are expressed as the mean ± SD for 3 independent measurements. Statistical significance was tested using a one sample *t*-test that control value is equal to 100% and is expressed as * *p* ˂ 0.05. ** *p* ˂ 0.01. *** *p* ˂ 0.001. N—number of measurements.

**Table 2:** Drug-induced monoamine oxidase (MAO-A and MAO-B) inhibition.

**MAO-A**										
**Drug**	**IC_50_** **(µM)**			**Hillslope**			**Residual Activity** **(rel.u.)**			**Inhibition**
agomelatine	8.20	±	1.41	3.40	±	1.72	0.190	±	0.070	partial
ketamine	10.36	±	8.28	1.50	±	1.83	0.259	±	0.214	partial
vortioxetine	7.33	±	1.10	1.76	±	0.61	0.109	±	0.050	partial
**MAO-B**										
agomelatine		-			-			-		none
ketamine	51.16	±	25.54	2.12	±	2.04	0.707	±	0.161	weak
vortioxetine	18.24	±	3.50	2.58	±	0.68	0.229	±	0.041	partial

The values are expressed as the mean ± SEM for 4 independent measurements. IC_50_ is half maximal inhibitory concentration.

**Table 3:** Correlation coefficients for agomelatine-induced changes in mitochondrial parameters.

Agomelatine	Complex I Activity		Complex IV Activity		Complex I-Linked ATP Kinetics		Complex II-Linked ATP Kinetics	
	**r**	**N**	**r**	**N**	**r**	**N**	**r**	**N**
complex IV activity	0.01	11	-	-	-	-	-	-
complex I-linked ATP kinetics	−0.12	7	* 0.87	7	-	-	-	-
complex II-linked ATP kinetics	−0.76	6	−0.47	6	* 0.81	6	-	-
total H_2_O_2_ content	0.12	8	0.48	8	−0.77	6	-	-
complex I-linked respiration	−0.12	13	0.17	4	0.86	3	−0.59	6

The Pearson correlation coefficient (r) was used as a measure of linear correlation between mitochondrial parameters measured at various concentrations of antidepressants and statistical significance and it is expressed as * *p* ˂ 0.05. H_2_O_2_—hydrogen peroxide, N—number of measurements. Respiratory parameters unaffected by drugs were not included in the correlation analysis.

**Table 4:** Correlation coefficients for vortioxetine-induced changes in mitochondrial parameters.

Vortioxetine	Complex I Activity		Complex II+III Activity		Complex IV Activity		Complex I-Linked ATP Content		Complex II-Linked ATP Kinetics	
	**r**	**N**	**r**	**N**	**R**	**N**	**r**	**N**	**r**	**N**
complex II+III activity	*** 0.91	19	-	-	-	-	-	-	-	-
complex IV activity	0.41	10	*** 0.91	11	-	-	-	-	-	-
complex I-linked ATP content	* 0.69	11	** −0.78	11	−0.55	10	-	-	-	-
complex II-linked ATP kinetics	0.11	8	−0.48	8	−0.55	8	0.03	8	-	-
total ROS content	0.73	7	* 0.86	7	0.74	7	−0.42	7	−0.39	6
complex I-linked respiration	0.46	11	*** 0.86	12	* 0.67	9	−0.27	8	* 0.83	8

The Pearson correlation coefficient (r) was used as a measure of linear correlation between mitochondrial parameters measured at various concentrations of antidepressants and statistical significance and it is expressed as * *p* ˂ 0.05. ** *p* ˂ 0.01. *** *p* ˂ 0.001. ROS—reactive oxygen species, N—number of measurements. Respiratory parameters unaffected by drugs were not included in the correlation analysis.

## Data Availability

Not applicable.

## Abbreviations

5-HT	serotonin
AGO	agomelatine
AMPA	α-amino-3-hydroxy-5-methyl-4-isoxazolepropionic acid
ATP	adenosine triphosphate
BDNF	brain-derived neurotrophic factor
CS	citrate synthase
cyt *c*	cytochrome *c*
ETC	electron transport chain
FDA	Food and Drug Administration
H2O2	hydrogen peroxide
KET	ketamine
MDD	major depressive disorder
MDH	malate dehydrogenase
MPTP	1-methyl-4-phenyl-1,2,3,6-tetrahydropyridine
NADH	nicotinamide adenine dinucleotide
NMDA	*N*-methyl-d-aspartate
OXPHOS	oxidative phosphorylation
PFC	prefrontal cortex
ROS	reactive oxygen species
SSRIs	selective serotonin reuptake inhibitors
TrkB	tropomyosin receptor kinase B
VOR	vortioxetine
WHO	World Health Organization
